# Environmental and biotic factors affecting freshwater snail intermediate hosts in the Ethiopian Rift Valley region

**DOI:** 10.1186/s13071-020-04163-6

**Published:** 2020-06-08

**Authors:** Beekam Kebede Olkeba, Pieter Boets, Seid Tiku Mereta, Mesfin Yeshigeta, Geremew Muleta Akessa, Argaw Ambelu, Peter L. M. Goethals

**Affiliations:** 1 Laboratory of Environmental Toxicology and Aquatic Ecology, Ghent University, Coupure Links 653, Building F, 9000 Ghent, Belgium; 2 Department of Environmental Health Science and Technology, Jimma University, P.O.Box 378, Jimma, Ethiopia; 3 Department of Environmental Health Science, Hawassa University, P.O.Box 1560, Hawassa, Ethiopia; 4 Provincial Centre of Environmental Research, Godshuizenlaan 95, 9000 Ghent, Belgium; 5 Departement of Statistics, College of Natural Science, Jimma University, P.O.Box 378, Jimma, Ethiopia

**Keywords:** Freshwater snails, Environmental and biotic factors, Generalized linear model

## Abstract

**Background:**

Knowledge of the distribution and habitat preference of freshwater snail intermediate hosts can provide information to initiate and set-up effective snail control programmes. However, there is limited research conducted on the factors driving the occurrence and abundance of freshwater snail intermediate hosts in the Ethiopian Rift Valley. Hence, in this study, we investigated how environmental and biotic factors influence the occurrence and abundance of the snail intermediate hosts in Ethiopian Rift Valley region.

**Methods:**

Data on freshwater snails, physico-chemical water quality parameters, physical characteristics of habitat, predators and competitors, and anthropogenic activity variables were collected from 174 sampling sites during the wet season of 2017 and 2018. Generalized linear models were used to identify the main environmental and biotic factors affecting the occurrence and abundance of the snail species.

**Results:**

It was found that *Bulinus globosus* (31.7%) was the most abundant snail species followed by *Lymnaea natalensis* (21.6%), *Lymnaea truncatula* (15.1%) and *Biomphalaria pfeifferi* (14.6%). Generalized linear models indicated that physico-chemical parameters (water temperature, turbidity, chlorophyll-*a*, dissolved oxygen, chemical oxygen demand, alkalinity, calcium, magnesium, nitrate and ammonia), physical habitat characteristics (water depth, canopy cover, macrophyte cover and substrate type) and biotic factors (abundance of predators and competitors) were found to be the main variables determining the occurrence and abundance of snail species in the Ethiopian Rift Valley region. In terms of anthropogenic activities, human settlement, farming, bathing and swimming, clothes washing, grazing, drainage of land, car washing, boating, fishing and silviculture were also important variables determining the occurrence and abundance of snail species in the region.

**Conclusions:**

The findings reported herein suggest that integrated snail control strategies should be considered to control snails *via* protection of water bodies from disturbance by anthropogenic activities. In this way, it is possible to reduce the concentration of organic matter and dissolved ions in aquatic ecosystems which are conducive for the presence of snails.
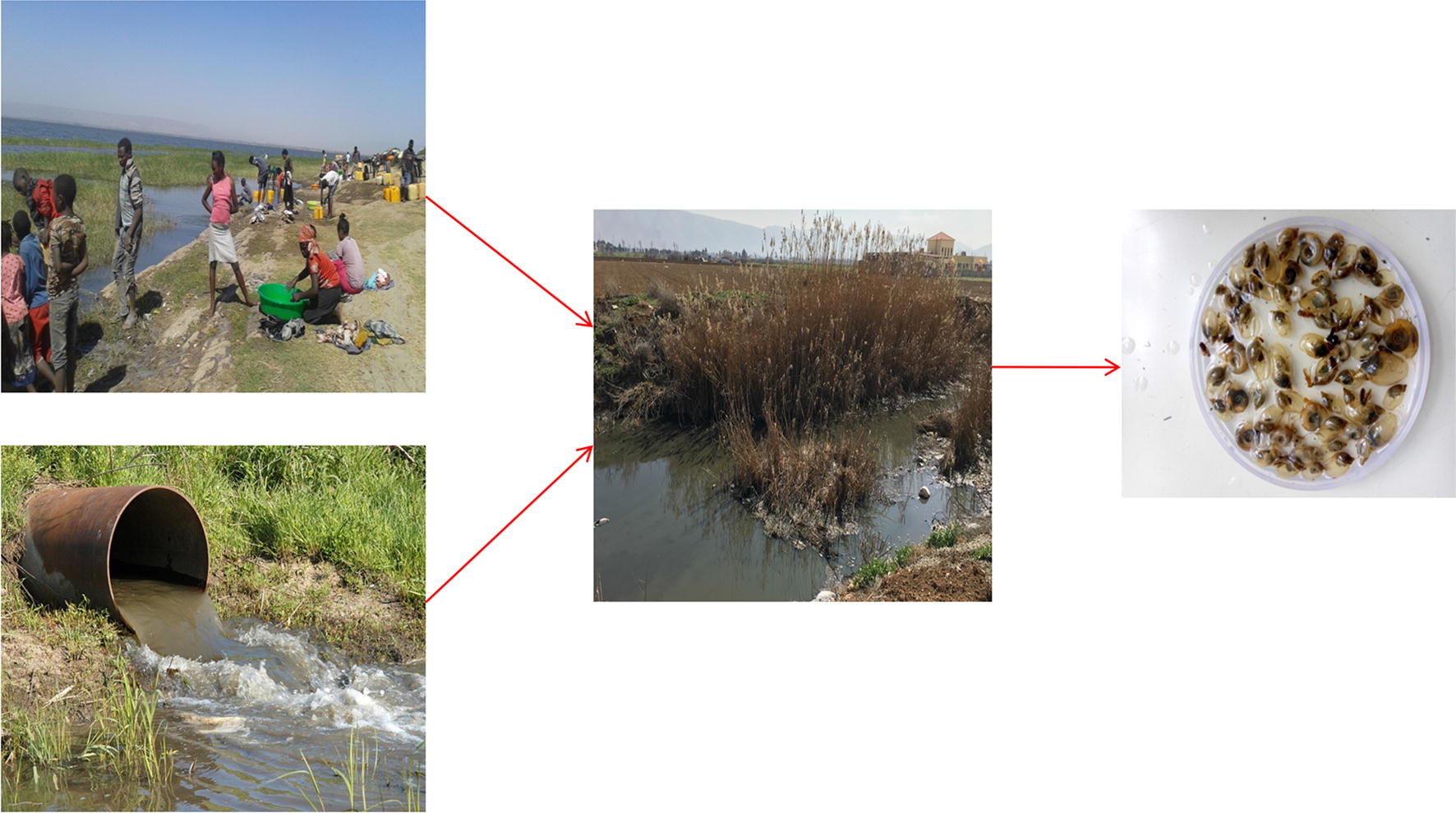

## Background

Snails are invertebrate animals of the class Gastropoda found in freshwater and other aquatic habitats around the world [1]. Approximately 5000 species of snails are found to inhabit different habitats worldwide [2]. Freshwater snails receive considerable attention as they are intermediate hosts of several trematodes that can cause diseases in humans and animals [3]. Among snail-borne diseases, schistosomiasis causes a major public health concern. It is estimated that 779 million people are at risk of schistosomiasis [4], with 250 million people at risk of infection, mainly in sub-Saharan Africa [5]. Schistosomiasis has a widespread distribution in several parts of Ethiopia usually at an altitude between 1200–2000 m above sea level [6], where an estimated 38.3 million people are living in schistosomiasis endemic areas [7]. In endemic areas, children have a greater risk of becoming infected as well as re-infected [8–10]. Fascioliasis is another important parasitic snail-borne disease in tropical and subtropical countries [11]. It has been estimated that 2.4 million people are infected worldwide with fascioliasis while 180 million people are at risk of infection [12]. The diseases are acquired through repeated contact with freshwater during washing, swimming or fishing [4, 5].

Transmission of snail-borne diseases depends on the distribution of specific freshwater snails that act as intermediate hosts and are the first step for a trematode parasite to reach the development stage to infect humans or animals [13, 14]. When parasite eggs are released into freshwater bodies with urine or feces, miracidia hatch and infect the intermediate host snails [15, 16]. In the snails, miracidium develops into a mother sporocyst. In schistosomes, the sporocyst develops into the second generation sporocysts, in which the infective larvae cercariae are formed. In some hermaphroditic trematodes (e.g. liver flukes), the mother sporocyst develops into rediae which produce cercariae [16, 17]. Once the cercariae are released into the water, they either penetrate the skin of the definitive host (e.g. schistosomes) or are ingested after encysting as metacercariae in or on edible plants or animals. After entering the definitive host, the schistosome larvae mature into adult worms in the blood vessels of the liver, intestine and bladder. The worms lay thousands of eggs that cause damage as they grow through tissues and consequently, infection occurs accordingly [17, 18]. Most malacologists studying freshwater snails focus mainly on snail species belonging to the genera *Biomphalaria*, *Bulinus* and *Lymnaea* which are known to serve as intermediate hosts of schistosomes and play a crucial role in the transmission of the disease in tropical and subtropical regions of the world [19]. Other freshwater snails that have no prominent medical or veterinary importance receive less or no attention. Generally, these are herbivores removing vegetation biomass, which may affect the standing crop and distribution of primary producers in an aquatic ecosystem [20]. In Africa, there are several snail species belonging to the genera *Biomphalaria*, *Bulinus* and *Lymnaea* that act as intermediate hosts of trematode parasites and that are of medical and veterinary importance [8].

A countrywide snail survey made in Ethiopia showed that planorbid (*Biomphalaria* spp.), bulinid and lymnaeid snails of medical and veterinary importance have a wide geographical distribution [21, 22]. It is recognized that two species of the genus *Biomphalaria* (*B. pfeifferi* and *B. sudanica*) are the sole intermediate hosts transmitting *Schistosoma mansoni* [23], whereas two species of the genus *Bulinus* (*Bu. abyssinicus* and *Bu. africanus*) are the intermediate hosts transmitting *Schistosoma heamatobium* [21]. The distribution of *B. sudanica* is restricted to the three Rift Valley areas of Ethiopia, along the shores of lakes Ziway and Abaya and the interface between Lake Hawassa and its tributary Tikur Wuha River [24], whereas *B. pfeifferi* has a ubiquitous distribution [21]. The distribution of *Bu. abyssinicus* and *Bu. africanus* is limited to the lowland areas, including the Awash and Wabe Shebele Valleys and along the Ethiopian-Sudan border [21]. *Lymnaea natalensis* and *L. truncatula* are the two snail species belonging to the genus *Lymnaea* found to transmit *Fasciola* parasites causing fascioliasis in humans [25, 26] and ruminant animals in Ethiopia [27, 28] and other African countries [29, 30]. Identifying factors that influence the distribution and habitat preference of the snail intermediate hosts is critical to snail-borne disease prevention and control efforts. In Africa, several ecological studies have shown that biotic and abiotic factors affect the distribution and habitat preference of freshwater snails [31–35]. Similarly, in Ethiopia, a number of studies have been carried out on the ecology of freshwater snails. These studies suggested that local environmental and biotic factors determine the occurrence and abundance of freshwater snails in each aquatic habitat due to environmental heterogeneity among ecological zones [22, 23, 35]. As in many other geographical regions in the country, the endemicity of snail-borne diseases has long been established in the Ethiopian Rift Valley [6, 36, 37]. However, there has been little research conducted on the factors driving the occurrence and abundance of freshwater snail intermediate hosts in the Ethiopian Rift Valley. Therefore, this study aimed to (i) determine the local distribution and diversity of freshwater snail intermediate hosts and (ii) examine the influence of environmental and biotic factors on their occurrence and abundance in the Ethiopian Rift Valley region. The findings of this study could be useful for the development of appropriate preventive and control measures against snail intermediate hosts in the Ethiopian Rift Valley.

## Methods

### Study area and location of sampling sites

**Fig. 1:**
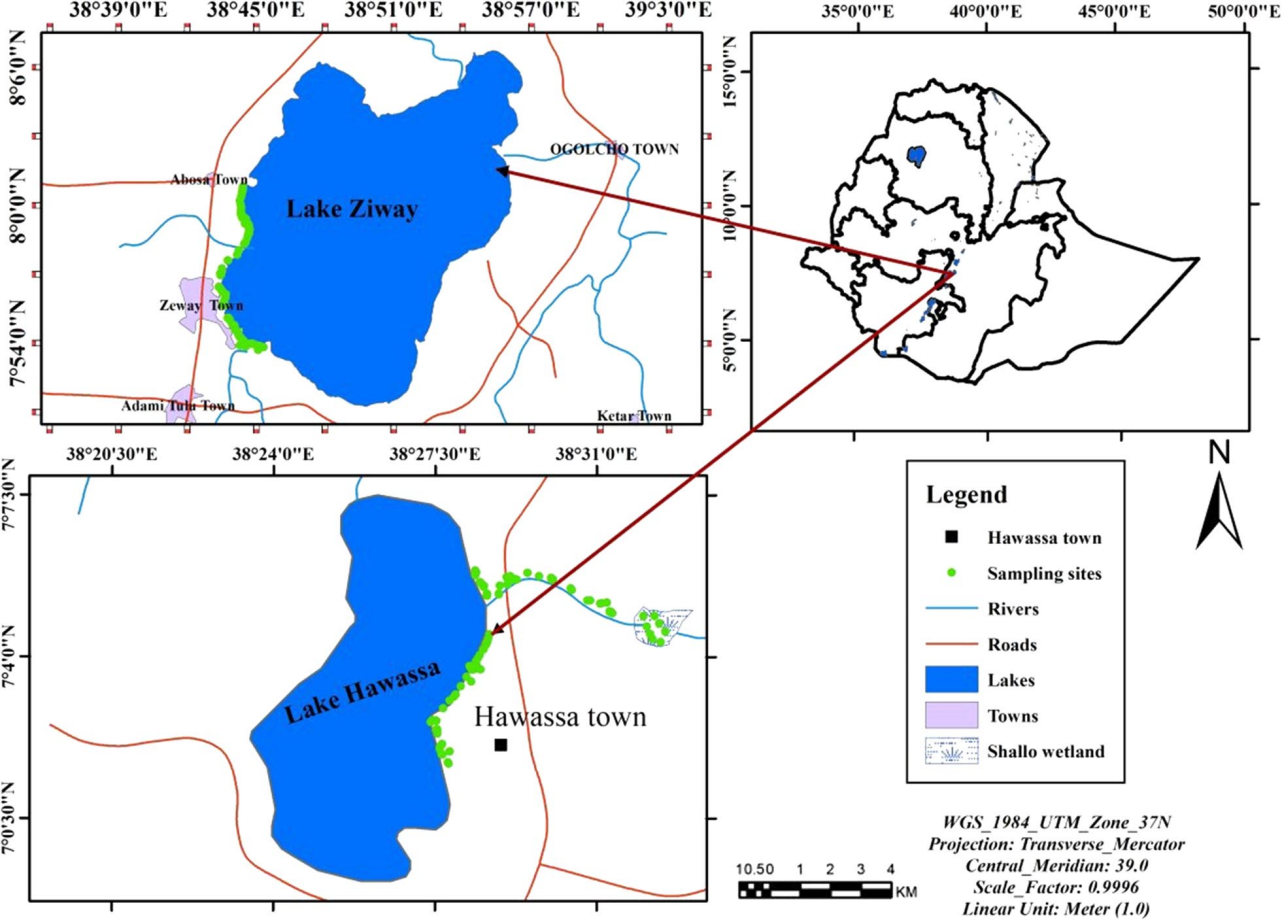
Map of the study area and the locations of the sampling sites (green dots) in the Ethiopian Rift Valley. Lake Hawassa and Shallo wetland correspond to the tropical rain climate zone, and Lake Ziway corresponds to the warm temperate rainy climate zone

Data was collected from two spatially distinct agro-ecological zones, warm temperate rainy and tropical rainy climate zones, which are located in the Ethiopian Rift Valley (Fig.1). These two studied zones are 125 km apart. In the warm temperate rainy climate zone, sampling was carried out along the shores of Lake Ziway which is situated between latitudes 07°51″N and 08°07″N and longitudes 38°43″E and 38°50″E at an elevation of 1636 m above sea level [38]. The mean annual rainfall and temperature of the area are 837 mm and 19.3 °C, respectively [39]. In the tropical rainy climate zone, sampling was carried out along the shores of Lake Hawassa and in Shallo wetland where the Tikur Wuha River drains before it joins Lake Hawassa. Lake Hawassa is situated between latitudes 06°58″N and 07°14″N and longitudes 38°22″E and 38°28″E at an elevation of 1685 m above sea level [40]. The area receives a mean annual rainfall of 950 mm and has a mean annual temperature of 19.8 °C [39]. The aquatic habitats surveyed from both agro-ecological zones are surrounded by local communities, and they serve as an important source of water for surrounding rural communities for various purposes such as domestic use, irrigation, livestock watering, fishing, recreation and alike.

**Fig. 2:**
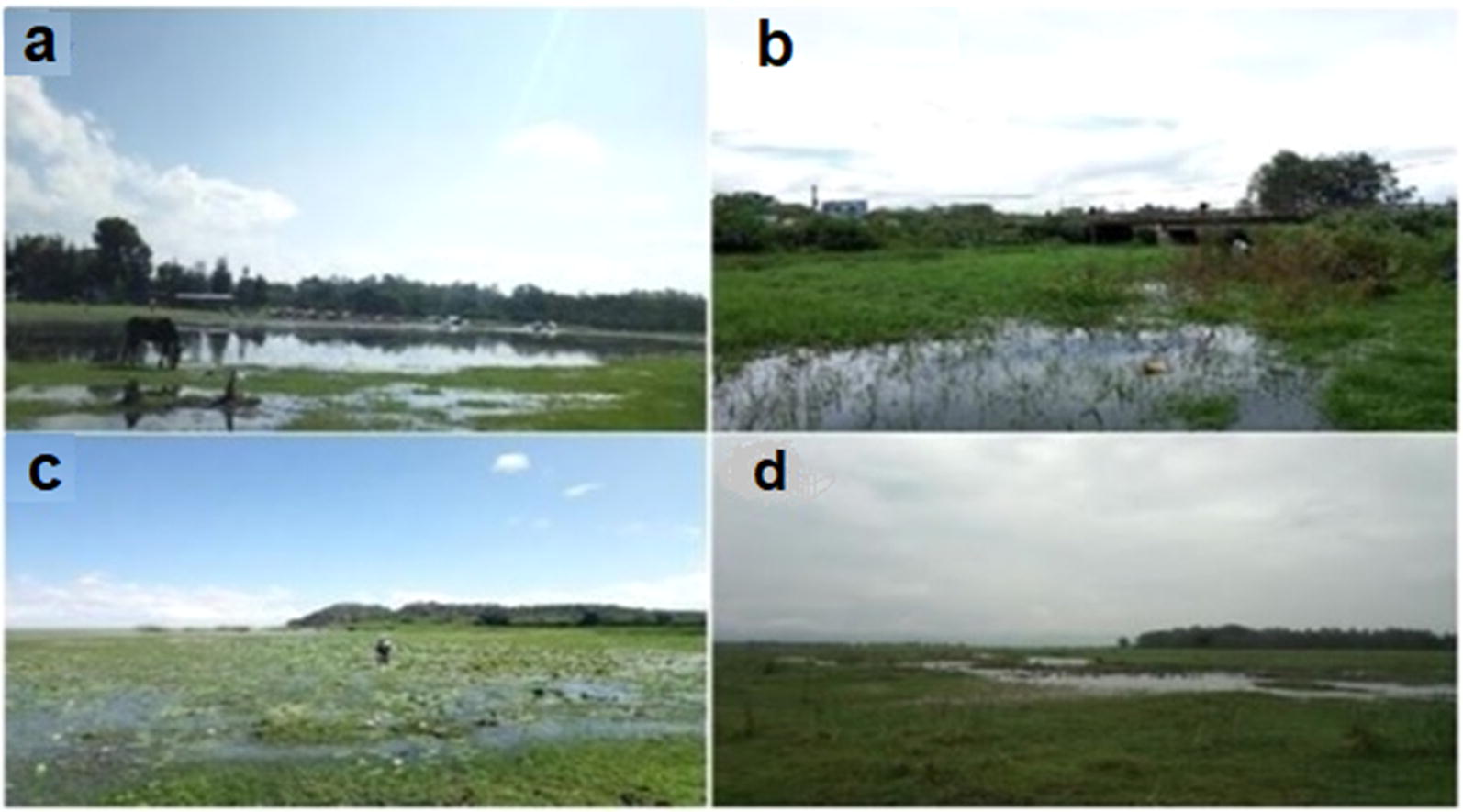
Pictures taken at sampling sites in the Ethiopian Rift Valley: Lake Hawassa (**a**, **b**); Lake Ziway (**c**); and Shallo wetland (**d**)

Field sampling was carried out during two consecutive years, both during the wet season (November, 2017 and 2018). Sampling sites were selected from a range of levels of accessibility to anthropogenic activities and existing variations in ecological characteristics of the habitats (Fig. 2).

### Environmental factors

Physico-chemical water quality measurements were performed both onsite during sampling and in the laboratory. Water temperature, pH, day-time dissolved oxygen and electrical conductivity were measured onsite using a multi-probe meter (HQ40d Single-Input Multi-parameter Digital Meter; Hach Company, Loveland, USA). Chlorophyll-*a* was measured onsite using a hand-held fluorometer (Aqua Fluor; Turner Designs, San Jose, USA). Turbidity was measured using a turbidity meter (Wag-WT3020; Halma PLC Company, Amersham, UK). In addition, water samples were collected from each sampling site in polyethylene bottles for analysis of other physico-chemical parameters. Afterwards, the samples were transported (in the dark) to the laboratory in an ice cooled box. Concentrations of total suspended solids, chemical oxygen demand, nitrate, ammonia, total phosphorus, orthophosphate, chloride, alkalinity, total hardness, calcium and magnesium were determined according to the standard method [41] at the laboratory of the Environmental Health Science and Technology of Jimma University, Ethiopia.

Physical characteristics of snail habitats, such as water depth, transparency and ambient temperature were measured at each sampling site using the United States Environmental Protection Agency habitat assessment protocol [42]. Water depth was measured using a graduated stick calibrated in centimeters. Transparency of water was determined with a Secchi disk 30 cm in diameter attached to a calibrated cord. Ambient temperature was measured using a mercury-in-glass thermometer. Percentage of aquatic macrophyte (emergent, submerged and floating) cover was visually estimated by a simple estimation of the proportion of the site covered by aquatic plants within a 500 m stretch considering the sampling site as the center. The percentage of the aquatic macrophyte cover was classified into five groups: very low (< 10%); low (10–35%); moderate (35–65%); high (65–90%); and very high (> 90%) [43]. Canopy cover was estimated visually based on the percentage of shade [44]. At each sampling site, substrate was carefully assessed by observation and classified into grass, silt, detritus, sand, gravel, boulders and bedrock [45]. Presence or absence (1/0) of anthropogenic activities in and around each sampling site was assessed by observation following the method used by Mereta et al. [46].

A hand-held global positioning system (GPS) unit was used to record geographical coordinate readings at each sampling site. The map of the study area showing the locations of sampling sites was created using the Geographic Information System (GIS) software ArcGIS10.3.

### Freshwater snails survey

Snails were collected using a standard scoop net with a mesh size of 300 µm supported by a metal frame [47]. Water samples were collected for the determination of physico-chemical parameters at the sampling sites where snails were collected. At each sampling site, all substrates were thoroughly searched to collect snails. Sampling time was fixed at 30 min per site. Sampling area per site was approximately 5 m^2^, whereas lengths of 10 m along the Lake shoreline and wetland were sampled. Afterwards, snails collected from each sampling site were transferred to plastic vials containing 80% ethanol to transport to the laboratory of the Department of Environmental Health Science and Technology, Jimma University, for identification. In the laboratory, each snail (except for specimens of the genera *Physa*, *Ferrisia* and *Burnupia*) was identified to species level according to the shell morphology using the field guide to African freshwater snails [48] and individuals were counted to determine the number of each snail species collected per sampling site.

### Predators and competitors survey

To collect data on invertebrate predators and competitors of the freshwater snail species, macroinvertebrates were sampled from the same sampling site where snails were collected. Macroinvertebrate sampling was carried out using a standard hand-held rectangular drag (20 × 30 cm) with a cutting metal frame covered with a 300 µm mesh net. Macroinvertebrate sampling was carried out for 10 min over a distance of 10 m by disturbing the bottom sediment by foot and by sweeping the kick net between macrophytes to dislodge the macroinvertebrates [49]. Samples of macroinvertebrates collected from each sampling site were sorted in the field, placed in labeled plastic vials containing 80% ethanol and transported to the laboratory for identification. In the laboratory, macroinvertebrates were identified to the family level based on morphology using a stereomicroscope (10×) and identification key [50] and then categorized according to functional feeding groups: predators, scrapers, gatherer-collectors, filterer-collectors, and shredders [51]. Afterwards, scrapers and macroinvertebrates belonging to the family Physidae [52] were considered as competitors of snails, whereas invertebrates such as Psychodidae [53], Dytiscidae [54], Hydrophilidae [55], Belostomatidae [56], Odonata [57] and Glossiphoniidae [58] were considered as predators of snails. Presence or absence (1/0) and abundance of predators and competitors were considered as biotic factors to assess the occurrence and abundance of freshwater snail species.

### Statistical analysis

Data exploration and regression modeling were performed using R software (Version 3.5.1) [59]. A Shapiro–Wilk normality test showed that data on abundance of each snail species did not show a normal distribution (*P* < 0.05). Therefore, a non-parametric Kruskal–Wallis ANOVA was used to test whether significant differences in the abundance of snail species existed between the agro-ecological zones, and in relation to presence/absence of predators and competitors. All statistical tests were performed at the significance level of 0.05.

Generalized linear models were used to identify the variables affecting the occurrence and abundance of the snail species. Logistic regression models were used to predict the occurrence of the snail species and zero-inflated Poisson regression models were used to predict the abundance. Because a large number of zero values reduce the power of the test statistic [60], only snail species with frequency of occurrence greater than 20% were used for regression analysis. Therefore, five snail species (i.e. *B. pfeifferi*, *B. sudanica*, *L. natalensis*, *L. truncatula* and *Bu. globosus*) were selected for regression analysis in this study.

During the preliminary analysis, the dataset was explored to detect outliers and collinearity among predictor variables to decrease uncertainty of the model [61]. To find a set of predictor variables that does not contain collinearity, Spearman’s rank order correlation coefficient was determined to generate a matrix of pairwise correlations between all predictor variables. As correlation coefficients only show pairwise correlations, we calculated variance inflation factors (VIFs) to assess which predictor variables are collinear and should be dropped before starting the analysis. This procedure continued until no further collinearity existed [61].

A stepwise backward selection procedure was followed to build the model starting from the full model. The model with the lowest Akaike information criterion (AIC) value was retained as the optimal model [61]. The goodness-of-fit of the models was assessed using the relation between the residuals and predictor variables and the normality of the residuals was tested using a QQ-plot (probability plot). Retained models were only considered reliable if no relations between the residuals and the predictor variables were visually observed and residuals were normally distributed [61].

## Results

### Occurrence and abundance of freshwater snails

A total of 2700 individual snails were collected from the 174 sampling sites. All collected snails were of the subclass Pulmonata which were grouped into 4 families: Planorbidae, Lymnaeidae, Physidae and Ancylidae. The snails were encountered in 137 sampling sites (79%).

The most abundant family was Planorbidae (*n* = 1494) which accounted for 55.3% of the total number of snails followed by the family Lymnaeidae, Physidae and Ancyclidae accounting for 36.6%, 8% and 0.48% of the total number of snails, respectively. The spatial distribution of the snail species shows that the tropical rainy climate zone had a higher relative abundance (57%) compared to the warm temperate rainy climate zone (43%). The frequency of occurrence of snails was higher in the warm temperate rainy climate zone accounting for 80% of the total sampling sites compared to the tropical rainy climate zone (77.5%). In addition, the warm temperate rainy climate zone supported higher snail richness (7 species and 3 genera) compared to the tropical rainy climate zone (7 species). Planorbidae and Lymnaeidae families were represented in both agro-ecological zones, whereas Physidae and Ancyclidae were collected only from the warm temperate rainy climate zone. The frequency of occurrence and abundance of the freshwater snail species collected during the study period are presented in Table 1.

**Table 1:** Abundance and frequency of occurrence (%) of the freshwater snail species in aquatic habitats located in the Ethiopian Rift Valley

Family	Genus	Species	Tropical rainy climate zone (*N* = 89)	Warm temperate climate zone (*N* = 85)	Overall
			*n* (%)	*n* (%)	*n* (%)
Planorbidae	*Biomphalaria*	*B. sudanica*	105 (26)	80 (21)	185 (24)
		*B. pfeifferi*	252 (47)	143 (25)	395 (36)
	*Bulinus*	*Bu. globosus*	638 (33)	217 (58)	855 (45)
		*Bu. forskalii*	16 (1)	2 (2)	18 (2)
		*Bu. truncatus*	24 (4)	17 (6)	41 (5)
Lymnaeidae	*Lymnaea*	*L. truncatula*	384 (51)	23 (9)	407 (30)
		*L. natalensis*	118 (36)	464 (53)	582 (44)
Physidae	*Physa*	*Physa* sp.		204 (11)	204 (5)
Ancylidae	*Ferrissia*	*Ferrissia* sp.		7 (1)	7 (1)
	*Burnupia*	*Burnupia* sp.		6 (2)	6 (1)

Among all genera of snail species collected, *Bu. globosus* was the most abundant snail species accounting for 32% of the total number of snails and it was encountered in 45% of the sampling sites. The species was mainly found in the tropical rainy climate zone. *Lymnaea natalensis* was the second most abundant snail species accounting for 22% of the total number of snails. It was recorded at 44% of the sampling sites and most of which were encountered in the warm temperate rainy climate zone. *Biomphalaria pfeifferi* and *B. sudanica* were collected from both the tropical rainy climate and warm temperate rainy climate zones and were recorded at 36% and 24% of the sampling sites, respectively. *Burnupia* was the least abundant snail genus collected from 1% of the sampling sites. A Kruskal–Wallis test indicated that the tropical rainy climate zone supported a significantly higher abundance of *B. pfeifferi* (*χ*^2^ = 9.71, *df* = 1, *P* = 0.0018), *L. truncatula* (*χ*^2^ = 36.47, *df* = 1, *P* < 0.0001), *Bu. globosus* (*χ*^2^ = 7.89, *df* = 1, *P* = 0.0049) and a higher abundance of the total number of snails (*χ*^2^ = 5.37, *df* = 1, *P* = 0.0204). A significantly higher abundance of predators (*χ*^2^ = 11.19, *df* = 1, *P* = 0008) was encountered in the tropical rainy climate zone, whereas a significantly higher abundance of competitors (*χ*^2^ = 13.30, *df* = 1, *P* = 0.0003) was observed in the warm temperate rainy climate zone.

### Factors affecting the occurrence of freshwater snail species

In the present study, the logistic regression model included water depth, turbidity, emergent macrophyte cover and human settlement as important factors determining the occurrence of *B. pfeifferi* and the model explained 23.4% of the variation (Additional file 1: Table S1). The occurrence of *B. pfeifferi* increased with increasing water depth, but decreased with increasing turbidity. Emergent macrophyte cover showed a positive association with regard to the occurrence of *B. pfeifferi* in the presence of human settlements (Additional file 1: Table S1). The logistic regression model determining the occurrence of *B. sudanica* selected water temperature, concentration of dissolved oxygen, submerged macrophyte cover and human settlements as determining factors (13.7% of variance explained) (Additional file 1: Table S1). The occurrence of *B. sudanica* increased with increasing water temperature, but decreased with increasing concentration of dissolved oxygen. Based on model output, submerged macrophyte cover promotes the occurrence of *B. sudanica* at sites situated near human settlements.

Water temperature, calcium concentration, magnesium concentration and agro-ecological zone were selected by the logistic regression model as important factors determining the occurrence of *L. truncatula* explaining 82.8% of the variation (Additional file 1: Table S1). The model demonstrated that increasing calcium concentration promotes the occurrence of *L. truncatula*, while increasing magnesium concentration and water temperature restrict its occurrence. *Lymnaea truncatula* is more likely to occur in the warm temperate rainy climate zone (Additional file 1: Table S1). There was no variable selected by the logistic regression model to explain the occurrence of *L. natalensis*.

According to the logistic regression model, water depth, alkalinity, chloride concentration, substrate type, agro-ecological zone and fishing explained the occurrence of *Bu. globosus* (36.7% of variance explained) (Additional file 1: Table S1). Based on the output of the model we found that increasing water depth and alkalinity positively influences the occurrence of *Bu. globosus*, while increasing chloride concentration leads to a decrease in the occurrence of *Bu. globosus*. *Bulinus globosus* frequently occurred in the warm temperate rainy climate zone, but was less likely to occur at sites with silt and in the presence of fishing activity (Additional file 1: Table S1).

### Factors affecting the abundance of freshwater snail species

According to the zero-inflated Poisson regression model, the abundance of *B. pfeifferi* was positively correlated with physico-chemical water quality parameters such as water temperature and chemical oxygen demand, but negatively correlated with turbidity and dissolved oxygen (Additional file 2: Table S2). A higher abundance of *B. pfeifferi* was found at sites with submerged macrophyte cover and emergent macrophyte cover and at sites characterized by the presence of anthropogenic activities (i.e. human settlements, farming and clothes washing). The species was found at lower abundances at sites where bathing and swimming, drainage of land and car washing took place.

The output of the model showed that the abundance of *B. sudanica* increased with increasing water temperature, alkalinity and canopy cover, but decreased with increasing dissolved oxygen concentration, chlorophyll-*a* and chloride concentration in the presence of human settlements. *Biomphalaria sudanica* was abundant at sites characterized by submerged macrophyte cover (Additional file 2: Table S2). Abundance of *L. natalensis* increased with increasing alkalinity and competitor abundance, but decreased with increasing ammonia concentration and dissolved oxygen (Additional file 2: Table S2). A zero-inflated Poisson regression model revealed that *L. natalensis* was found at higher abundances at sites where grazing by cattle was observed. The abundance of *L. truncatula* was positively related to nitrate concentration and magnesium concentration, but negatively related to water temperature, chlorophyll-*a*, ammonia concentration and calcium concentration (Additional file 2: Table S2). Anthropogenic activities (i.e. clothes washing and boating) reduced the abundance of *L. truncatula*, but car washing and the presence of a silviculture promoted the abundance of *L. truncatula* (Additional file 2: Table S2).

Water temperature, water depth, alkalinity, nitrate concentration, chloride concentration, ammonia concentration, predator abundance, canopy cover, floating macrophyte cover, emergent macrophyte cover, substrate type, agro-ecological zone and fishing were selected by the model as the main variables determining the abundance of *Bu. globosus* (Additional file 2: Table S2). Abundance of *Bu. globosus* increased with increasing water temperature, water depth, nitrate concentration, alkalinity concentration, predator abundance, but decreased with increasing chloride concentration, ammonia concentration and canopy cover (Additional file 2: Table S2). *Bulinus globosus* was found at higher abundances at sites with floating macrophyte cover in the presence of silt, but was less abundant at sites with emergent macrophyte cover and where detritus was present (Additional file 2: Table S2).

## Discussion

This ecological investigation of gastropods focused on identifying environmental and biotic factors significantly affecting the occurrence and abundance of freshwater snail intermediate hosts in the Ethiopian Rift Valley. This study revealed that the habitat preference of freshwater snail species depends on physico-chemical water quality parameters, physical characteristics of habitat, biological factors and anthropogenic activities.

Regression analysis between physico-chemical water quality parameters and the occurrence and abundance of snail species showed that *B. pfeifferi*, *B. sudanica*, *L. truncatula* and *Bu. globosus* were abundant at sites with a relatively higher water temperature. This might be due to the fact that a higher temperature increases the food availability [62]. Higher temperature also crucially increases the snail metabolic rate and therefore increases the size of the snail population by reducing the duration of the development periods [35, 63]. On the other hand, *L. truncatula* showed an opposite trend and less likely occurred at higher temperatures in this study. This could be due to the species being more sensitive to thermal stress compared to the other species [64]. Previous studies pointed out that water temperature is one of the important factors determining the occurrence and abundance of snail species [26, 62, 64, 65]. This study revealed that *B. pfeifferi*, *B. sudanica* and *L. natalensis* were more abundant at sites with a low dissolved oxygen. *Biomphalaria pfeifferi* was more abundant at sites with a high chemical oxygen demand, whereas it was less likely to occur and less abundant at sites with a high turbidity. A positive correlation of snail species to low values of dissolved oxygen and chemical oxygen demand could be explained by the ability of snail species to occupy water bodies with a high content in organic matter [66]. Outputs of the models indicated that *B. sudanica*, *L. natalensis* and *Bu. globosus* were abundant at a high water alkalinity, whereas *B. sudanica* and *Bu. globosus* were less abundant at sites with a high chloride concentration. Our findings match with the results of the study by Oloyede et al. [33], who recorded high abundance of snails in alkaline water. Calcium and magnesium concentrations were also identified as important factors significantly affecting the occurrence and abundance of *L. truncatula*. The ratio of calcium to magnesium is important for calcium uptake for the development of snail shells [67, 68]. A higher nitrate concentration is an indication of eutrophication and thus favours the occurrence of the snails [69].

Previous studies reported that water depth was an important ecological factor determining the distribution of snail species [33, 35, 67]. Similarly, in our study, we found a positive relationship between water depth and the occurrence of both *B. pfeifferi* and *Bu. globosus* and a negative relationship between water depth and the occurrence of *L. truncatula*. On the other hand, the abundance of *B. pfeifferi* was positively associated to water depth. Moreover, we found that *B. sudanica* was abundant at sites characterized by a high canopy cover, which supplies the snails with shade and shelter [70]. However, *Bu. globosus* was less abundant at sites characterized by a high canopy cover which is probably related to indirect effects of canopy cover on snails through a negative impact on the ability of sunlight to reach the bottom and decrease primary production, which represents the food supply for snails [71]. A negative correlation between chlorophyll-*a* content and the abundance of both *B. sudanica* and *L. truncatula* observed in our study is in line with previous studies [69, 72, 73]. Chlorophyll-*a* is necessary for the productivity of plants and algae in a water body which is used as a food source for snails. However, the level of chlorophyll-*a* measured in this study, 14.03 μg/l, is above 10 μg/l that support high concentrations of harmful algae to aquatic organisms [74]. The increase in presence of *B. pfeifferi* and the increase in abundance of *B. sudanica* and *Bu. globosus* at sites with a high macrophyte cover observed in our study, indicate that food availability and the accessibility of aquatic weeds as a suitable surface on which egg masses of snails are deposited play an important role [21, 62, 75, 76]. On the contrary, the decrease in abundance of *Bu*. *globosus* at sites with macrophyte cover could be due to the fact that a dense emergent macrophyte cover prevents oxygen which promotes growth of snails [67].

A positive relationship between *Bu. globosus* abundance and predator abundance and between *L. natalensis* abundance and competitor abundance observed in the present study might be due to the presence of macrophyte cover that may suppress the predation and competition activities [70]. This finding is consistent with the earlier reports by Dejenie et al. [77], who reported that predator abundance was positively associated with the increasing number of aquatic invertebrates. This finding, however, contradicts that of Yigezu et al. [35], who found that the abundance of snails decreases as the abundance of predators increases (Tables 2, 3).

**Table 2:** Descriptive statistics for the continuous predictor variables used to assess the occurrence and abundance of freshwater snail species in the Ethiopian Rift Valley

Predictor variable	Tropical rainy climate zone			Warm temperate rainy climate zone		
	Range	Mean ± SD	Median	Range	Mean ± SD	Median
Altitude (m)	1655–1699	1678.9 ± 7	1681	1618–1645	1633.3 ± 5.3	1634
Water depth (m)	0.2–2.0	0.7 ± 0.4	0.6	0.1–1.2	0.5 ± 0.2	0.5
Transparency (m)	0.1–0.6	0.2 ± 0.1	0.2	0.1–0.8	0.3 ± 0.1	0.2
Ambient temperature (°C)	18–30	25 ± 2.9	26	16–30	23.7 ± 2.9	24
Water temperature (°C)	14.2–27.6	22 ± 3	22.2	15.2–30.2	21.9 ± 3.8	21.8
Dissolved oxygen (mg/l)	0.1–16	4.7 ± 2.7	4.5	0.3–21.5	6.5 ± 3.6	6.2
Oxygen saturation (%)	1.1–209	66 ± 38.7	60.4	4.4–349	96.1 ± 62.2	91.4
Chemical oxygen demand (mg/l)	7.9–50.4	19.8 ± 8.1	18.4	3.4–41.3	15.7 ± 7.3	16.2
Electrical conductivity (μS/cm)	137.3–2015	757 ± 431.1	902	233–3680	893 ± 590.7	700
pH	5.4–9.6	8.2 ± 0.9	8.9	3.1–9.8	7.5 ± 1.6	8.1
Turbidity (NTU)	2.8–247	24.6 ± 43.5	14.1	1.8–159	32 ± 34.8	13.9
Chlorophyll-*a* (µg/l)	12.7–41.4	15.5 ± 4.5	14.1	12.6–33.1	14.9 ± 2.8	13.9
Total suspended solids (mg/l)	90.8–687.2	268.8 ± 174	200.8	87.2–759.6	170.1 ± 111.7	145.6
Chloride (mg/l)	5–87	34.7 ± 18.7	38	16–252.9	37.1 ± 33.6	26
Nitrate (mg/l)	0–130.3	7 ± 17.3	0.5	0–95.3	8.4 ± 17.8	0.5
Ammonia (mg/l)	0–5.1	0.7 ± 0.9	0.4	0–5.1	0.8 ± 1.1	0.3
Total phosphorus (mg/l)	0–24.7	1.9 ± 3.4	1.0	0–11.1	1.2 ± 1.5	0.8
Orthophosphate (mg/l)	0–23.9	1.1 ± 2.8	0.4	0–7.8	0.5 ± 0.9	0.3
Alkalinity (mg/l)	78–896	381.4 ± 185.9	404	164–1414	376.1 ± 230.5	304
Total hardness (mg/l)	22–184	51.5 ± 23.4	46	52–316	116.4 ± 45.1	112
Calcium (mg/l)	14–114	32.4 ± 18.4	26	32–196	71.6 ± 34.6	60
Magnesium (mg/l)	0–76	19.1 ± 12.8	18	4–190	44.8 ± 29.9	44
Canopy cover (%)	0–100	21.5 ± 32.2	0	0–100	29.5 ± 35.7	10
Predator abundance (*n*)	0–153	19.4 ± 23.5	10	0–43	9.2 ± 9.4	1
Competitor abundance (*n*)	0–5	0.3 ± 0.8	0	0–79	2.9 ± 11.8	0

*Abbreviation*: SD, standard deviation

**Table 3:** The frequency and percentages of the categorical predictor variables used to assess the occurrence and abundance freshwater snail species in the Ethiopian Rift Valley

Predictor variable	Category	Tropical rainy climate zone		Warm temperate rainy climate zone	
		Frequency	Percentage	Frequency	Percentage
Emergent macrophyte cover	Very low	32	34	11	13
	Low	13	14	7	8
	Moderate	15	16	25	29
	High	19	20	13	15
	Very high	10	11	29	34
Submerged macrophyte cover	Very low	44	47	40	47
	Low	45	48	44	52
	Moderate	0	0	0	0
	High	0	0	0	0
	Very high	0	0	1	1
Floating macrophyte cover	Very low	30	32	30	35
	Low	38	41	40	47
	Moderate	9	10	7	8
	High	6	7	3	4
	Very high	6	75	5	6
Substrate type	Grass	50	56	72	85
	Silt	25	28	7	8
	Detritus	14	16	6	7
Predators occurrence	Absent	6	6	6	7
	Present	83	86	79	85
Competitors occurrence	Absent	87	90	81	87
	Present	2	2	4	4
Farming	Absent	71	80	62	73
	Present	18	20	23	27
Solid waste dumping	Absent	82	92	69	81
	Present	7	8	16	19
Grazing	Absent	52	58	34	40
	Present	37	42	51	60
Silviculture	Absent	73	82	64	75
	Present	16	18	21	25
Drainage of land	Absent	80	90	73	86
	Present	9	10	12	14
Open defecation	Absent	86	97	77	91
	Present	3	3	8	9
Effluent discharge	Absent	88	99	83	98
	Present	1	1	2	2
Fishing	Absent	75	84	70	82
	Present	14	16	15	18
Bathing and swimming	Absent	80	90	72	85
	Present	9	10	13	15
Clothes washing	Absent	81	91	71	84
	Present	8	9	14	17
Car washing	Absent	85	96	84	99
	Present	4	5	1	1
Boating	Absent	82	92	84	99
	Present	7	8	1	1
Settlement	Absent	63	71	79	93
	Present	26	29	6	7

According to the survey, it was observed that the occurrence and abundance of snail species is associated with anthropogenic activities such as human settlement, farming, bathing and swimming, clothes washing, grazing, drainage of land, car washing, boating, fishing and silviculture in the study area. The increased presence and high abundance of snail species in habitats disturbed by anthropogenic activities could be due to the high concentration of organic matter and dissolved ions which are conducive for snail species [30, 34, 35, 78–80]. The results of the present study show that *Bu. globosus* was likely to occur, and more abundant, in the warm temperate rainy climate zone. However, *L. truncatula* frequently occurred, and was less abundant, in the warm temperate rainy climate zone compared to the tropical rainy climate zone. Differences in ecological and climatic conditions between the agro-ecological zones may be responsible for this result.

### Implication for snail control

Results of this study revealed that snail species occurred frequently and were abundant in habitats disturbed by anthropogenic activities such as settlement, farming, clothes washing, car washing, grazing, silviculture, and bathing and swimming. Hence, integrated snail control strategies should be considered as a priority to reduce the presence and density of freshwater snail intermediate hosts and thus to control the spread of snail-borne diseases at a local scale. Protection of water bodies from disturbance by anthropogenic activities (i.e. through regulation of human settlement in the areas) may be a good strategy to reduce the concentration of organic matter and dissolved ions in aquatic ecosystem, which are conducive for presence of snails. However, contact with cercariae-infested water is a decisive factor related to the risk of acquiring snail-borne diseases. Hence, a safe water supply may be an alternative approach to reduce the likelihood of cercariae infecting a final host. On the other hand, even if safe water supplies reduce such water contact, they may not completely prevent it. Therefore, a reduction in infection could be attempted through behavioral changes, such as boiling water before drinking it, preventing animals from getting to the water bodies, and the use of commercial soap and a locally available soapberry endod (*Phytolacca dodecandra*) as a detergent during water contact, which appears to provide some protection from infection.

## Conclusions

Generalized linear models indicated that physico-chemical water quality parameters, physical habitat characteristics and biotic factors were found to be the main variables determining the occurrence and abundance of snail species in the Ethiopian Rift Valley region. This study also revealed that snail species frequently occurred and were abundant in habitats disturbed by anthropogenic activities. Therefore, the findings reported herein suggest that integrated snail control strategies should be considered to control snails *via* protection of water bodies from disturbance by anthropogenic activities. In this way, it is possible to reduce the concentration of organic matter and dissolved ions in aquatic ecosystem which are conducive to the presence of snails. This study may be used as baseline for further studies on trematode infections in snail intermediate hosts from these water bodies and epidemiology of schistosomiasis among lakeshore communities to ascertain whether active transmission is occurring within these areas.

## Supplementary information

**Additional file 1: Table S1.** Output of the logistic regression model.

**Additional file 2: Table S2.** Output of the zero-inflated Poisson regression model.

## Data Availability

Data supporting the conclusions of this article are included within the article and its additional files. The dataset generated and/or analyzed during the present study is available from the corresponding author.

## Abbreviation

NTU: nephelometric turbidity unit
