# Supplementary material for: Environmental and biotic factors affecting freshwater snail intermediate hosts in the Ethiopian Rift Valley region
Source: Parasit Vectors. 2020 Jun 8;13:292. doi: 10.1186/s13071-020-04163-6 (PMC7282061; doi:10.1186/s13071-020-04163-6)
Supplement: Supplementary file 1 — Additional file 1: Table S1. Output of the logistic regression model. [file 13071_2020_4163_MOESM1_ESM.docx]

**Additional file 1:Table S1.** Output of the logistic regression model in the generalized linear model to model the occurrence of snail species

| Snail species | Variables |  | Estimate | Standard error | Z value | Pr(>\|z\|) |
| --- | --- | --- | --- | --- | --- | --- |
| *B. pfeifferi* | Intercept |  | -7.412 | 2.618 | -2.832 | 0.005 ** |
|  | Water depth |  | 1.712 | 0.670 | 2.556 | 0.011 * |
|  | Turbidity |  | -0.015 | 0.007 | -2.176 | 0.030 * |
|  | Emergent macrophyte cover | <10% |  |  |  |  |
|  |  | 10-35% |  |  |  |  |
|  |  | 35-65% |  |  |  |  |
|  |  | 65-90% | 2.327 | 1.058 | 2.198 | 0.030 * |
|  |  | >90% |  |  |  |  |
|  | Settlement |  | 1.292 | 0.540 | 2.394 | 0.017 * |
| *B. sudanica* | Intercept |  | -6.958 | 2.666 | -2.609 | 0.009 ** |
|  | Water temperature |  | 0.266 | 0.110 | 2.407 | 0.016 * |
|  | Dissolved oxygen concentration |  | -0.324 | 0.102 | 3.168 | 0.002 ** |
|  | Sub-merged macrophyte cover | <10% |  |  |  |  |
|  |  | 10-35% | 1.958 | 0.851 | 2.300 | 0.021 * |
|  |  | 35-65% |  |  |  |  |
|  |  | 65-90% |  |  |  |  |
|  |  | >90% |  |  |  |  |
|  | Settlement |  | 1.348 | 0.498 | 2.704 | 0.007 ** |
| *L. truncatula* | Intercept |  | 3.109e+00 | 7.688e+00 | 0.404 | 0.686 |
|  | Water temperature |  | -6.928e-01 | 2.513e-01 | -2.757 | 0.006 ** |
|  | Calcium |  | 8.426e-02 | 3.321e-02 | 2.537 | 0.011 * |
|  | Magnesium |  | -1.160e-01 | 5.246e-02 | -2.211 | 0.027 * |
|  | Agro-ecological zone | Warm temperate rainy | 7.174e+00 | 2.289e+00 | 3.134 | 0.002 ** |
|  |  | Tropical rainy |  |  |  |  |
| *Bu. globosus* | Intercept |  | -2.566 | 2.495 | -1.028 | 0.304 |
|  | Water depth |  | 2.229 | 0.801 | 2.785 | 0.005 ** |
|  | Alkalinity |  | 0.006 | 0.002 | 2.438 | 0.015 * |
|  | Chloride |  | -0.042 | 0.020 | -2.103 | 0.035 * |
|  | Habitat type | Wetland | -3.469 | 1.104 | -3.143 | 0.002 ** |
|  |  | Lake |  |  |  |  |
|  | Agro-ecological zone | Warm temperate rainy | 1.144 | 0.550 | 2.079 | 0.038 * |
|  |  | Tropical rainy |  |  |  |  |
|  | Fishing |  | -1.450 | 0.588 | -2.552 | 0.011 * |
|  | Substrate type | Grass |  |  |  |  |
|  |  | Silt | -2.632 | 0.914 | -2.881 | 0.004 ** |
|  |  | Detritus |  |  |  |  |

Signif.codes: 0 ‘***’ 0.001 ‘**’ 0.01 ‘*’ 0.05 ‘.’ 0.1 ‘ ’ 1
