# Supplementary material for: Environmental and biotic factors affecting freshwater snail intermediate hosts in the Ethiopian Rift Valley region
Source: Parasit Vectors. 2020 Jun 8;13:292. doi: 10.1186/s13071-020-04163-6 (PMC7282061; doi:10.1186/s13071-020-04163-6)
Supplement: Supplementary file 2 — Additional file 2: Table S2. Output of the zero-inflated Poisson regression model. [file 13071_2020_4163_MOESM2_ESM.docx]

Additional file 2: Table S2. Output of the zero-inflated Poisson regression model in the generalized linear model to model the abundance of snail species

| **Snail species** | **Variables** |  | **Estimate** | **Standard error** | **Z value** | **Pr(>\|z\|)** |
| --- | --- | --- | --- | --- | --- | --- |
| ***B. pfeifferi*** | Intercept |  | 5.541 | 2.770 | 0.000 | 0.000*** |
|  | Water temperature |  | 0.399 | 0.114 | 3.955 | 0.000*** |
|  | Turbidity |  | -0.025 | 0.007 | -3.760 | 0.000*** |
|  | Dissolved oxygen saturation |  | -0.019 | 0.006 | -3.346 | 0.001*** |
|  | Chemical oxygen demand |  | 0.061 | 0.028 | 2.164 | 0.030* |
|  | Sub-merged macrophyte cover | <10% |  |  |  |  |
|  |  | 10-35% |  |  |  |  |
|  |  | 35-65% | 2.756 | 0.844 | 3.267 | 0.001** |
|  |  | 65-90% |  |  |  |  |
|  |  | >90% |  |  |  |  |
|  | Emergent macrophyte cover | <10% |  |  |  |  |
|  |  | 10-35% | 1.724 | 0.683 | 2.526 | 0.012* |
|  |  | 35-65% |  |  |  |  |
|  |  | 65-90% |  |  |  |  |
|  |  | >90% |  |  |  |  |
|  | Drainage of land |  | -1.925 | 0.776 | -2.480 | 0.013* |
|  | Settlement |  | 1.182 | 0.479 | 2.467 | 0.014* |
|  | Farming |  | 1.416 | 0.554 | 2.557 | 0.011* |
|  | Cloth washing |  | 1.716 | 0.842 | 2.040 | 0.041* |
|  | Car washing |  | -2.693 | 1.229 | -2.192 | 0.028* |
|  | Bathing and swimming |  | -1.990 | 0.911 | -2.184 | 0.029* |
| ***B. sudanica*** | Intercept |  | -10.092 | 3.179 | -3.184 | 0.002** |
|  | Water temperature |  | 0.538 | 0.138 | 3.90e+00 | 0.000*** |
|  | Dissolved oxygen concentration |  | -0.515 | 0.130 | -3.969 | 0.000*** |
|  | Alkalinity |  | 0.003 | 0.002 | 1.443 | 0.149* |
|  | Chloride |  | -0.040 | 0.020 | -1.987 | 0.047* |
|  | Chlorophyll-*a* |  | -0.194 | 0.093 | -2.081 | 0.037* |
|  | Canopy cover |  | 0.017 | 0.008 | 2.081 | 0.030* |
|  | Settlement |  | 2.181 | 0.588 | 3.716 | 0.000 |
|  | Sub-merged macrophyte cover | <10% |  |  |  |  |
|  |  | 10-35% | 4.154 | 0.992 | 4.19e+00 | 0.000*** |
|  |  | 35-65% |  |  |  |  |
|  |  | 65-90% |  |  |  |  |
|  |  | >90% |  |  |  |  |
| ***L. natalensis*** | Intercept |  | -0.030 | 0.351 | -0.080 | 0.934 |
|  | Alkalinity |  | 0.002 | 0.002 | 3.770 | 0.000*** |
|  | Dissolved oxygen saturation |  | -0.005 | 0.002 | -2.090 | 0.037* |
|  | Ammonia |  | -0.617 | 0.198 | -3.120 | 0.002** |
|  | Competitors abundance |  | 0.052 | 0.016 | 3.290 | 0.001** |
|  | Habitat type | Wetland | -3.550 | 0.821 | -4.320 | 0.000*** |
|  |  | Lake |  |  |  |  |
|  | Grazing |  | 0.702 | 0.309 | 2.270 | 0.023* |
|  | Bathing and swimming |  | 2.117 | 0.445 | 4.760 | 0.000*** |
| ***L. truncatula*** | Intercept |  | 19.686 | 3.356 | 5.870 | 4.50e-09*** |
|  | Water temperature |  | -0.391 | 0.098 | -3.970 | 7.10e-05*** |
|  | Chlorophyll-*a* |  | -0.461 | 0.116 | -3.990 | 6.70e-05*** |
|  | Nitrate |  | 0.149 | 0.033 | 4.460 | 8.10e-06*** |
|  | Ammonia |  | -2.784 | 0.723 | -3.850 | 0.000*** |
|  | Calcium |  | -0.063 | 0.018 | -3.430 | 0.000*** |
|  | Magnesium |  | 0.061 | 0.018 | 3.290 | 0.001*** |
|  | Habitat type | Wetland | -6.277 | 1.552 | -4.050 | 5.20e-05*** |
|  |  | Lake |  |  |  |  |
|  | Cloth washing |  | -2.135 | 0.784 | -2.720 | 0.006** |
|  | Car washing |  | 4.443 | 1.257 | 3.540 | 0.000*** |
|  | Boating |  | -2.242 | 0.717 | -3.130 | 0.002** |
|  | Silviculture |  | 1.669 | 0.505 | 3.300 | 0.001*** |
|  | Agro-ecological climate zone | Warm temperate rainy | -4.063 | 0.866 | -4.690 | 2.70e-06*** |
|  |  | Tropical rainy |  |  |  |  |
|  | Substrate type | Grass |  |  |  |  |
|  |  | Silt |  |  |  |  |
|  |  | Detritus | -2.731 | 0.605 | -4.520 | 6.30e-06*** |
| ***Bu. globosus*** | Intercept |  | -4.222 | 2.132 | -1.980 | 0.048* |
|  | Water temperature |  | 0.174 | 0.075 | 2.300 | 0.021* |
|  | Water depth |  | 1.711 | 0.574 | 2.980 | 0.003** |
|  | Chloride |  | -0.038 | 0.015 | -2.620 | 0.009** |
|  | Ammonia |  | -0.983 | 0.263 | -3.020 | 0.003** |
|  | Nitrate |  | 0.051 | 0.016 | 3.210 | 0.001** |
|  | Alkalinity |  | 0.008 | 0.002 | 4.210 | 0.000*** |
|  | Canopy cover |  | -0.014 | 0.007 | -2.080 | 0.038* |
|  | Predators abundance |  | 0.021 | 0.011 | 1.980 | 0.048* |
|  | Emergent macrophyte cover | <10% |  |  |  |  |
|  |  | 10-35% |  |  |  |  |
|  |  | 35-65% |  |  |  |  |
|  |  | 65-90% |  |  |  |  |
|  |  | >90% |  |  |  |  |
|  |  | >90% | -2.000 | 0.653 | -3.060 | 0.002** |
|  | Floating macrophyte cover | 10-35% |  |  |  |  |
|  |  | 35-65% |  |  |  |  |
|  |  | 65-90% | 2.194 | 0.770 | 2.850 | 0.004** |
|  |  | >90% |  |  |  |  |
|  |  | >90% |  |  |  |  |
|  | Substrate type | Grass |  |  |  |  |
|  |  | Silt | 1.425 | 0.466 | 3.050 | 0.002** |
|  |  | Detritus | -1.652 | 0.674 | -2.450 | 0.014* |
|  | Habitat type | Wetland | -4.358 | 1.056 | -4.130 | 0.000*** |
|  |  | Lake |  |  |  |  |
|  | Agro-ecological climate zone | Warm temperate rainy | 1.532 | 0.453 | 3.380 | 0.001*** |
|  |  | Tropical rainy |  |  |  |  |
|  | Fishing |  | -1.721 | 0.463 | -3.720 | 0.000*** |

Signif.codes: 0 ‘***’ 0.001 ‘**’ 0.01 ‘*’ 0.05 ‘.’ 0.1 ‘ ’ 1
